# Functional outcomes and complications of intramedullary fixation devices for Midshaft clavicle fractures: a systematic review and meta-analysis

**DOI:** 10.1186/s12891-020-03256-8

**Published:** 2020-06-22

**Authors:** Paul Hoogervorst, Tess van Dam, Nico Verdonschot, Gerjon Hannink

**Affiliations:** 1 Department of Orthopedic Surgery, Radboud University Medical Center, P.O. Box 9101, 6500 HB Nijmegen, The Netherlands; 2 Department of Orthopedic Surgery, University of Minnesota, 2450 Riverside Avenue South, Suite R200, Minneapolis, MN 55454 USA; 3 BAAT Medical BV, Hengelo, The Netherlands; 4 Department of Biomechanical Engineering, University of Twente, Enschede, The Netherlands; 5 Department of Operating Rooms, Radboud University Medical Center Nijmegen, Enschede, The Netherlands

**Keywords:** Clavicle, Fracture, Complication, Survival, Failure, Function, Intramedullary

## Abstract

**Background:**

An alternative to the current gold standard in operative treatment of displaced midshaft clavicle fractures (DMCF) using plate osteosynthesis, is internal fixation by means of intramedullary fixation devices. These devices differ considerably in their specifications and characteristics and an evaluation of their clinical results is warranted. The aim of this systematic review is to generate an overview of functional outcomes and complications in the management of DMCF per available intramedullary device.

**Methods:**

A systematic review was conducted to identify all papers reporting functional outcomes, union rates and/or complications using an intramedullary fixation device for the management of midshaft clavicle fractures. Multiple databases and trial registries were searched from inception until February 2020. Meta-analysis was conducted based on functional outcomes and type of complication per type of intramedullary fixation device. Pooled estimates of functional outcomes scores and incidence of complications were calculated using a random effects model. Risk of bias and quality was assessed using the Cochrane risk of bias and ROBINS-I tools. The confidence in estimates were rated and described according to the recommendations of the GRADE working group.

**Results:**

Sixty-seven studies were included in this systematic review. The majority of studies report on the use of Titanium Elastic Nails (TEN). At 12 months follow up the Titanium Elastic Nail and Sonoma CRx report an average Constant-Murley score of 94.4 (95%CI 93–95) and 94.0 (95%CI 92–95) respectively (GRADE High). The most common reported complications after intramedullary fixation are implant-related and implant-specific. For the TEN, hardware irritation and protrusion, telescoping or migration, with a reported pooled incidence 20% (95%CI 14–26) and 12% (95%CI 8–18), are most common (GRADE Moderate). For the Rockwood/Hagie Pin, hardware irritation is identified as the most common complication with 22% (95%CI 13–35) (GRADE Low). The most common complication for the Sonoma CRx was cosmetic dissatisfaction in 6% (95%CI 2–17) of cases (GRADE Very low).

**Conclusion:**

Although most studies were of low quality, good functional results and union rates irrespective of the type of device are found. However, there are clear device-related and device-specific complications for each. The results of this systematic review and meta-analysis can help guide surgeons in choosing the appropriate operative strategy, implant and informing their patient.

**Level of Evidence:**

IV

## Background

Clavicle fractures are common fractures with an incidence reported of 59.3 per 100,000 person years [1]. Historically, these fractures were predominantly treated non-operatively. However, it has been reported that surgical treatment of displaced mid-shaft clavicle fractures (DMCF) leads to better union rates, improved early functional outcomes, and increased patient satisfaction [2–4]. The current gold standard in operative treatment is Open Reduction Internal Fixation (ORIF) using plates and screws. An alternative to this technique is internal fixation using intramedullary fixation devices. These devices aim to reduce the DMCF in a minimally invasive manner and thereby improving cosmetic satisfaction and union rates while lowering infection rates [5]. There are multiple different intramedullary devices available. Some of these devices are made out of rigid stainless steel while others consist of flexible titanium alloys. Some are not fixated within the bone while others are fixated on either one or both sides of the midshaft clavicle fracture. Since these devices differ considerably in their specifications and characteristics the array and distribution of complications and functional outcomes may vary as well.

The aim of this systematic review is to generate an overview of functional outcomes and complications in the management of DMCF per available intramedullary devices.

## Methods

Electronic databases (PubMed, ScienceDirect, Embase and Cochrane) and clinical trial registries (ClinicalTrials.gov, controlled-trials.com (ISRCTN), Australian New Zealand Clinical Trials Registry (ANZCTR), Chinese Clinical Trial Registry (CCTR), EU Clinical Trials Register (EU-CTR) and The Netherlands National Trial Register (NTR)) were searched from their inception to February 2020. Keywords used to develop our search strategy were ‘clavicle’, ‘fracture’, ‘intramedullary fixation’. The detailed search strategy is described in Additional file 1.

### Inclusion criteria

All titles and abstracts were screened and study inclusion was decided on by two reviewers (PH/TvD). In case of discrepancy in study inclusion, disagreements were discussed until consensus on eligibility was reached. If disagreement persisted after discussion, consensus was met consulting GH. References of retrieved eligible articles were searched for supplementary studies. Studies meeting the following criteria were included:

- Studies describing the functional outcomes, with use of any type of intramedullary fixation for DMCF.
- Studies describing complications, with use of any type of intramedullary fixation for DMCF.
- Only original studies were included.
- Studies written in English, Dutch, and German.
- Studies concerning skeletally mature patients.

Abstracts, theses, case reports, biomechanical studies, surgical technique papers, editorials, letters and conference proceedings were not included. Studies using Kirschner wires and screws were excluded. Studies concerning intramedullary fixation for open fractures, pathological fractures, multi-trauma patients, floating shoulders, non-unions or mal-unions were also excluded.

### Data extraction

Studies in the final study selection were divided into subgroups depending on type of implant and ranked according to their study design and level of evidence (Oxford Centre of Evidence Based Medicine) by 2 authors (PH, TvD). The level of evidence (LoE) rating is divided into 5 levels: level I indicates the highest evidence studies, level II high, level III moderate, level IV low and level V very low-evidence studies [6]. Disagreement between the reviewers concerning quality assessment was resolved by discussion.

Data from all included studies were extracted with respect to specific characteristics including title, author, year of publication, number of clavicles reported, type of fracture, intramedullary device used, length of follow-up, functional outcomes, and type and number of complications. Date were extracted and checked for accuracy by PH and TvD. Discrepancies were resolved by discussion. This study was conducted and reported in accordance with the reporting guidance provided in the Preferred Reporting Items for Systematic Reviews and Meta-Analyses (PRISMA) statement [7]. The protocol was prospectively registered in PROSPERO (CRD42018086518).

### Risk of bias and quality assessment

The Cochrane risk of bias tool was used for assessing risk of bias in randomized trials.

The risk of bias tool covers six domains of bias: selection bias, performance bias, detection bias, attrition bias, reporting bias, and other bias. Within each domain, assessments are made for one or more items, which may cover different aspects of the domain, or different outcomes [8].

The ROBINS-I tool was used for assessing risk of bias in non-randomized studies of interventions [9]. This tool assesses seven domains through which bias might be introduced. The first two domains, covering confounding and selection of participants into the study, address issues before the start of the interventions. The third domain addresses classification of the interventions themselves. The other four domains address issues after the start of interventions: biases due to deviations from intended interventions, missing data, measurement of outcomes, and selection of the reported result.

Publication bias was assessed only if 10 or more studies were included in the meta-analysis using funnel plots and Egger’s (for continuous outcomes) and Peters’ test (for proportions) for funnel plot asymmetry [10–12]. Sensitivity analyses were performed to assess the influence of study quality when there was more than 1 high quality study available according to the ROBINS-I.

The confidence in estimates were rated and described according to the recommendations of the GRADE working group as each outcome was assessed for potential risk of bias, inconsistency, imprecision, indirectness and publication bias [13].

### Data analysis

A meta-analysis was performed whenever three or more studies per intramedullary device that reported on a functional outcome or type of complication could be included.

Despite anticipated heterogeneity, the individual study proportions were pooled. Pooled estimates with their corresponding 95% confidence intervals were calculated using logit transformation (complications) or using untransformed data (functional outcome scores) within a random effects model framework. A continuity correction of 0.5 was applied if a study had an event probability of either 0 or 1. This continuity correction is used both to calculate individual study results with confidence limits and to conduct the meta-analysis. Heterogeneity of combined study results was assessed by I^2^, and its connected Chi-square test for heterogeneity, and the corresponding 95% confidence intervals were calculated. Restricted maximum likelihood was used to estimate the heterogeneity variance. 95% Prediction intervals were calculated to present the expected range of true effects in similar studies [14].

Statistical analyses were performed using R version 3.4.4 (R Foundation for Statistical Computing, Vienna, Austria) with package ‘meta’.

## Results

The search strategy retrieved 368 unique records. Subsequent selection procedure resulted in 75 eligible articles of which 67 studies could be included in this systematic review and 62 in the meta-analysis (Additional file 2). In total, 10 studies concerning the Rockwood (DePuy, Warsaw, IN, USA) and Hagie pin (Smith & Nephew, Memphis, TN, USA) were identified and included in the analysis (two level I, [15, 16] two level III [17, 18] and six level IV [19–24] studies). These devices were evaluated together since they are essentially the same; they both consist of the exact same stainless-steel pin, with a cancellous and machine thread end, and two nuts. The only difference between the two is that the Rockwood pin also has a trocar point on the machine thread end of the pin. Concerning the Titanium Elastic Nail (TEN) (Depuy Synthes, Warsaw, IN, USA or Stryker, Kalamazoo, MI, USA) the 43 studies that were incorporated in the analysis were comprised of seven level I, [25–31] eight level II, [32–39] eleven level III [40–50] and seventeen level IV [5, 51–66] studies. Another type of fixation described was the Sonoma CRx (Arthrex, Naples, FL, USA) for which 6 studies (three level I, [67–69] one level II, [70] one level III [71] and one level IV [72]) were identified. Less frequently described intramedullary fixation devices were the threaded titanium elastic nails (Kang Li Min Medical Devices Co. Ltd., Tianjin, China), [73–75] the Knowles pin (Zimmer Biomet, Warshaw, IN, USA) [76–79] and one study describing a second generation Titanium elastic nail (Puwei Medical Appliances Inc., Shanghai, China) [80]. Table 1 displays study characteristics including population description, type of intramedullary device, functional outcome scores, and type and number of complications.

**Table 1:** Study characteristics

						Functional Outcomes			Complications										
Author	Year	Level of Evidence	Study Design	Number of Patients	Clavicles	CMS(SD) at 12months	DASH(SD) at 12months	QuickDASH(SD) at 12months	Number of complications	Hardware irritation	Soft tissue problems	Hardware failure	Infection	Non-union	Protrusion/Telescoping/Migration	Delayedunion	Malunion	Pain	Cosmetic dissatisfaction
**RockwoodPin&HagiePin**																			
Strauss et al.	2007	4	RCS	16	16				8		3	2		0				1	
Judd et al.	2009	1	RCT	29	29				21	9		1	8	1		1			
Ferran et al.	2010	1	RCT	17	17	92.1(6)			4		1	1		0					
Mudd et al.	2011	4	RCS	18	18				16	3	3		2	3	2	1		1	
Kleweno et al.	2011	3	RCS	18	18				5	2	1	1	1	0					
Millett et al.	2011	4	RCS	51	51				15		5	2	2	5		1			
Payne et al.	2011	4	RCS	68	68				62	30		3	7	2		1		15	
Frye et al.	2012	4	RCS	17	17				11	7	1	2		0					
Marlow et al.	2012	4	RCS	70	70		5.9^a^		31	12	4		8	2				1	
Wenninger et al.	2013	3	RCS	33	33				3	2			1	0					
**TEN**																			
Jubel et al.	2002	2	PCS	65	65	96.9(3.3)			8		2			1	5				
Jubel et al.	2002	3	RCC	20	20	97(4)			0					0					
Jubel et al.	2003	3	RCS	55	58	97.9(3.3)			9	3	2		0	1	2				
Jubel et al.	2003	2	PCS	12	12	98.3(1.5)			0				0	0					
Jubel et al.	2005	2	PCC	26	26				20	8			0	0	2				
Kettler et al.	2005	4	RCS	55	55	81(7.1)			31	14	2		0	1	6		2	2	
Walz et al.	2006	2	PCS	35	35	98.1(1.3)			6	5			0	0	1				
Keener et al.	2006	4	RCS	24	24				13	6		2			1	1	3		
Kettler et al.	2007	4	RCS	87	87	84(9)	6.9(7.2)		23	4			0	2	4		7		4
Mueller et al.	2007	4	RCS	32	32	95(1.9)	5(2.3)		16	5		2	1	0	8				
Witzel	2007	2	RCT	35	35				0										
Hartmann et al.	2008	4	RCS	15	15	95.3(3.9)			4	4			0	0					
Frigg et al.	2009	4	RCS	34	34		1.5(3.2)		24	7		1		0	15			1	
Smekal et al.	2009	1	RCT	30	30	97.9(1.7)			10			2	0	0	7	1			
Liu et al.	2010	3	RCC	51	51	86.7(5.3)	13.5(3.9)		20	4		4	3	5			4		
Frigg et al.	2011	3	RCC	44	44		1.4(3.1)		14	5		1		1	6				
Chen et al.	2011	1	RCT	30	30	97(4.3)	2.74(3.6)		10	3		1	1	0	3				
Assobhi	2011	1	RCT	19	19	95.5(5.3)			4	3		0	0	0					1
Smekal et al.	2011	1	RCT	60	60	98(3.6)	0.5(1.8)		19	5		2	1	0	7	2			
Kadakia et al.	2012	4	RCS	38	38			6.7(3.4)	11	18			0	0	1				
Wijdicks et al.	2012	4	RCS	47	47				60	29		1	4	0	26			2	
Tarng et al.	2012	3	RCC	25	25	96(2)			4		4		0	0					
Chen et al.	2012	3	RCC	57	57	95(3.2)	4(4.4)		32	4		3	1	1	17				
Prokop et al.	2013	4	RCS	136	136	97(3)			1			1							
Langenhan et al.	2014	4	RCS	37	37	96.0(5.3)	3(5)		4				1	0	3				
Saha et al.	2014	2	PCC	34	34	93.5(4.4)			13	12			0	0					
Shokouh et al.	2014	4	RCS	12	13				0				0	0					
Braun et al.	2014	4	RCS	40	40	86.3(8.1)	5.5(6.9)		19	1	2			0	12				
Narsaria et al.	2014	2	PCC	33	33	94.6(3.2)			4			1	1	1					
Suresha et al.	2014	4	RCS	20	20	94.6^a^			0			0	0	0					
Lu et al.	2014	4	RCS	27	27	93,6(9)	6.2(11.1)		17	8		0	0	0	9				
Wang et al.	2015	3	RCC	25	25	93.8(8.9)	5.5(10.5)		12	5		0	0	0	5				
Andrade-Silva et al.	2015	1	RCT	25	25	91.8(8.8)	7.5(12.5)		10	10				1					
vanderMeijden et al.	2015	1	RCT	62	62	96.3(11.8)	3.9(10.2)		43	33									
Eden et al.	2015	2	PCC	24	24				5	1			1	1	2	1			
Mishra et al.	2016	3	PCC	73	73	96.8(2.3)			15	7			3	0	2	3			
Lechler et al	2016	3	RCC	36	36	87.7(10.7)	3.9(6.6)		12					3					
Fuglesang et al.	2017	1	RCT	60	60				36	19	4	2		1					
Govindasamy et al.	2017	4	RCS	54	54	97.8(1)			19	15			3	0	1	1			
Eickhoff et al.	2018	3	RCC	99	99				39	29	1			2	26				
Eisenstein et al.	2018	4	RCS	7	7				4	2	1				1				
Frima et al.	2018	4	RCC	34	34				20			4	0						
Zhang et al.	2019	3	RCC	37	37	97.3(13.7)			2			0	1				0		
**SonomaCRx**																			
Zehir et al.	2015	1	RCT	24	24			7.7(2.2)	8			1	0	0				3	4
King et al.	2015	2	PCS	47	47	90(13)	11(18)		3			2	1	0					
Zehir et al.	2015	4	RCS	17	17	94.3(2.8)	11.8(2.5)		2			1	1	0					
Calbiyik et al.	2016	1	RCT	35	35	92.9(4)		3.8(1.6)	5			2	1	0		1			1
ZehirS et al.	2016	3	RCC	33	33	94.3(5.3)			4			1	2	0				2	1
Kingetal.	2019	1	RCT	35	35	97(5)	5(6)		3		1	1	0						
**ThreadedPin**																			
Zenni et al.	1981	4	RCS	21	21				7			1	0		0				
Grassi et al	2001	3	RCC	40	40	82.9(8)			15				8	2		2			
Bi et al.	2015	2	PCS	45	45	96.5(9)	1.4(12.5)		20		19	1	0	0					
**KnowlesPin**																			
Chu et al	2002	4	RCS	78	78	92(13.8)			4			1				3			
Lee et al	2007	2	RCT	32	32	85(8.8)			0										
Lee et al.	2008	3	RCC	56	56				4	4									
Wu et al.	2013	4	RCC	337	337				19					19					
**2**^**nd**^**GenerationTEN**																			
Fu	2016	4	RCC	36	36	93.4(2.7)	2.5(1.6)		3	1					2				

*RCS* retrospective case series, *RCC* retrospective comparative cohort, *PCS* prospective case series, *PCC* prospective comparative cohort, *RCT* randomized clinical trail

^a^No range or SD reported

### Risk of bias assessment

The results of the Cochrane risk of bias tool are summarized in Table 2 and shows high risk of bias in domains 3 and 4 assessing performing and detection bias. The results of the ROBINS-I risk of bias assessment, summarized in Table 3 shows that the overall ROBINS-I score for most studies were subject to serious or critical risk of bias.

**Table 2:**
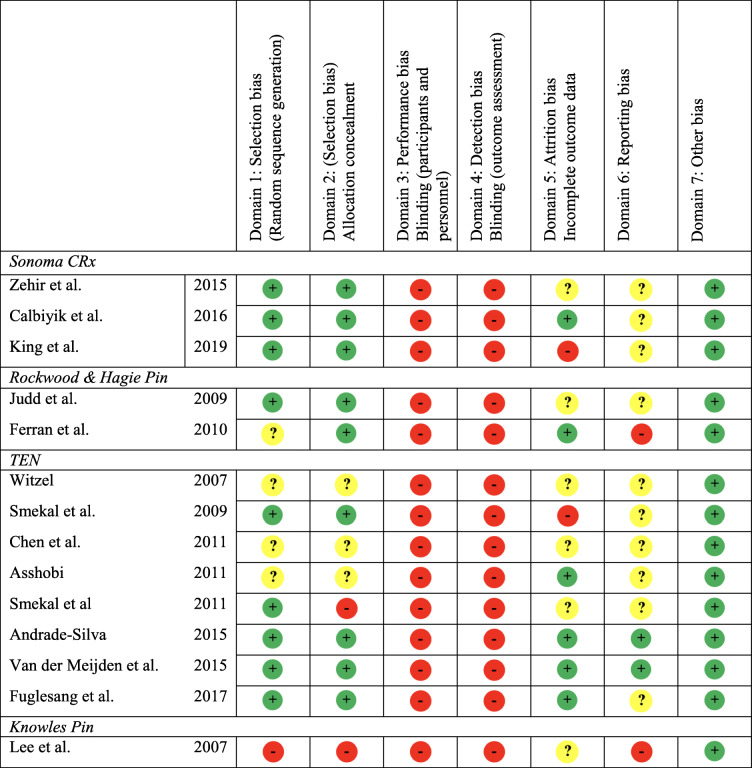
Cochrane risk of bias assessment of randomized trials

*Green* low risk, *Red* high risk, *Yellow* Unknown Risk

**Table 3:** ROBINS-I assessing risk of bias in non-randomized studies of interventions

Author	Year	Domain 1: Confounding	Domain 2: Selection of participants	Domain 3: Classification of intervention	Domain 4: Deviation from interventions	Domain 5: Missing data Domain	Domain 6: Measurement of outcomes	Domain 7: Selection of reported results	ROBINS-I overall
**Sonoma CRx**									
Zehir et al.	2015	2	2	2	1	2	2	2	2
King et al.	2015	3	3	2	1	2	2	2	3
Zehir et al.	2015	3	3	2	1	3	2	2	3
Calbiyik et al.	2016	2	1	1	1	1	2	2	2
Zehir S et al.	2016	3	2	2	1	2	2	3	3
**Rockwood Pin & Hagie Pin**									
Strauss et al.	2007	4	3	3	1	2	3	3	4
Judd et al.	2009	2	2	1	1	1	2	2	2
Ferran et al.	2010	2	2	2	1	1	2	2	2
Mudd et al.	2011	3	3	2	1	1	3	2	3
Kleweno et al.	2011	3	2	2	1	1	3	3	3
Millett et al.	2011	3	3	3	1	2	2	2	3
Payne et al.	2011	3	2	2	1	2	2	2	3
Frye et al.	2012	3	3	3	1	2	3	3	3
Marlow et al.	2012	3	3	2	1	2	2	2	3
Wenninger et al.	2013	3	2	2	1	2	3	2	3
**TEN**									
Jubel et al.	2002	2	2	1	1	2	2	2	2
Jubel et al.	2002	2	3	2	1	1	2	2	3
Jubel et al.	2003	3	3	2	1	2	2	3	3
Jubel et al.	2003	3	3	1	1	2	2	2	3
Jubel et al.	2005	2	3	1	1	1	2	2	3
Kettler et al.	2005	4	3	1	1	2	2	2	4
Walz et al.	2006	2	2	1	1	1	2	2	2
Keener et al.	2006	4	3	2	1	3	2	3	3
Kettler et al.	2007	3	3	2	1	2	2	2	3
Mueller et al.	2007	2	2	1	1	1	2	1	2
Witzel	2007	3	2	2	1	2	2	2	3
Hartmann et al.	2008	3	3	2	1	2	2	3	3
Frigg et al.	2009	3	2	1	2	2	2	3	3
Smekal et al.	2009	2	2	1	1	1	2	2	2
Liu et al.	2010	3	3	2	1	2	2	3	3
Frigg et al.	2011	2	2	1	1	3	2	2	3
Chen et al.	2011	2	2	1	1	1	2	2	2
Assobhi	2011	2	2	2	1	1	2	2	2
Smekal et al.	2011	2	2	1	1	1	2	2	2
Kadakia et al.	2012	4	3	2	1	2	3	2	4
Wijdicks et al.	2012	3	2	3	1	2	3	2	3
Tarng et al.	2012	3	3	3	1	2	2	2	3
Chen et al.	2012	3	3	2	1	2	2	2	3
Prokop et al.	2013	3	3	2	1	3	2	3	3
Langenhan et al.	2014	2	3	2	1	2	2	3	3
Saha et al.	2014	3	2	2	1	2	2	2	3
Shokouh et al.	2014	2	3	2	1	2	3	2	3
Braun et al.	2014	2	3	2	1	2	2	2	3
Narsaria et al.	2014	2	2	1	1	2	2	2	2
Suresha et al.	2014	3	3	2	1	2	2	2	3
Lu et al.	2014	2	3	1	1	2	2	2	3
Wang et al.	2015	2	3	1	1	2	2	2	3
Andrade-Silva et al.	2015	2	1	1	1	1	2	1	2
van der Meijden et al.	2015	2	1	1	1	1	2	1	2
Eden et al.	2015	3	2	2	1	2	2	2	3
Mishra et al.	2016	2	2	2	1	2	2	2	2
Lechler et al	2016	3	3	2	1	2	2	2	3
Fuglesang et al.	2017	2	2	1	1	2	2	2	2
Govindasamy et al.	2017	3	3	2	1	3	2	2	2
Eickhoff et al.	2018	2	2	1	1	2	2	2	2
Eisenstein et al.	2018	3	2	2	1	2	2	2	3
Frima et al.	2018	2	2	2	1	2	2	2	2
Zhang et al.	2019	2	3	2	1	3	3	3	3
**Threaded Pin**									
Zenni et al.	1981	4	4	2	1	2	3	2	4
Grassi et al	2001	3	3	2	1	2	2	2	3
Bi et al.	2015	2	2	2	1	2	2	2	2
**Knowles Pin**									
Chu et al	2002	3	3	2	3	3	2	3	3
Lee et al	2007	3	2	2	1	2	2	2	3
Lee et al.	2008	3	3	2	1	2	2	2	3
Wu et al.	2013	3	2	2	1	2	3	2	3

*1* low risk of bias, *2* moderate risk of bias, *3* serious risk of bias, *4* critical risk of bias

### Studies concerning the Rockwood pin and Hagie pin

All studies identified concerning these devices described an identical surgical technique. All pins were removed after union between 6 and 20 weeks through a secondary surgical intervention. Average follow-up of the studies ranged between 6 months and 7 years. The functional outcome scores reported were heterogeneous and therefore not comparable. Only two studies reported a Constant-Murley (92.1 ± 6) [15] or DASH (5.9) [19]. Other functional outcome scores reported were the Oxford Shoulder Score (45.2 ± 2.3), [15] L’Insalata (95.5 ± 7.3), [16] and ASES (88.6 and 89) [20, 24].

#### Meta-analysis:

**Fig. 1:**
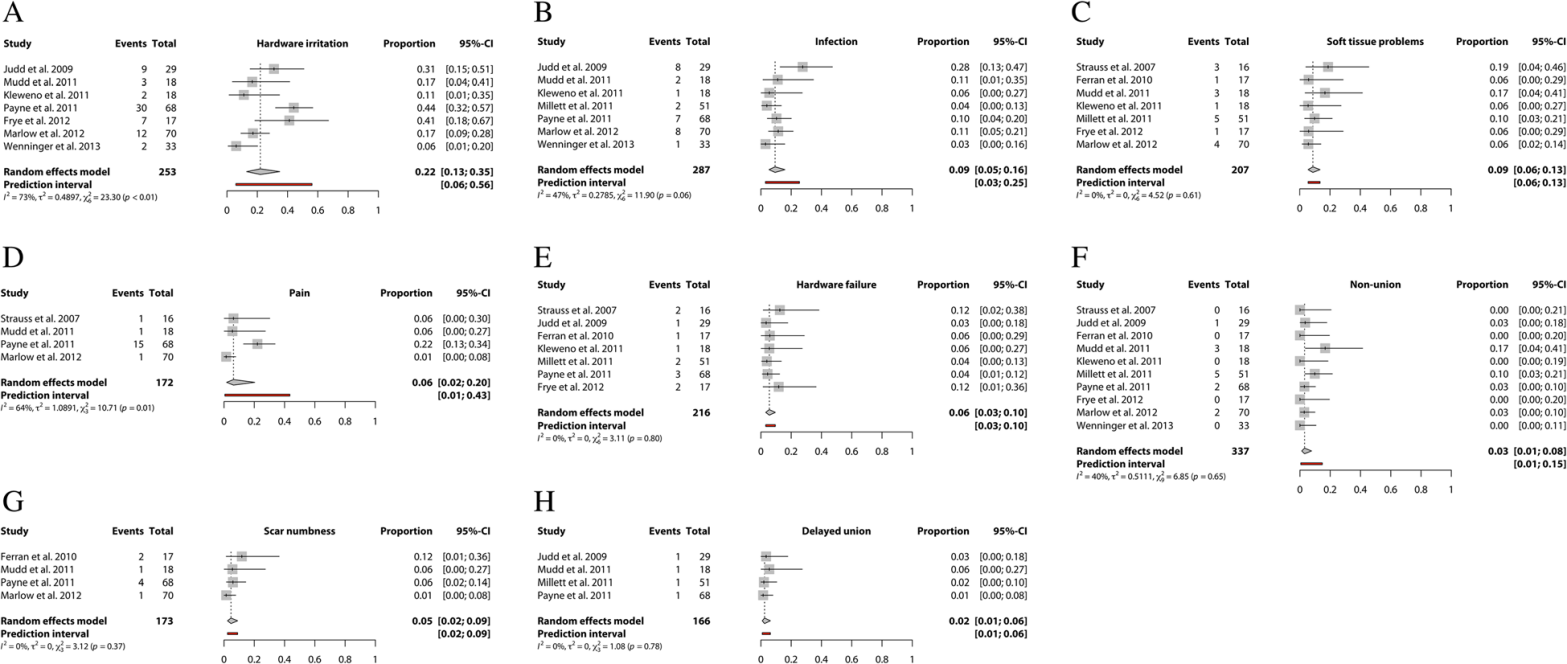
Forest plots of the included studies using the Rockwood and Hagie Pin reporting on (**a**) hardware irritation, (**b**) infection, (**c**) soft tissue problems, (**d**) persistent pain, (**e**) hardware failure, (**f**) nonunion, (**g**) scar numbness, and (**h**) delayed union. Forest plots display the mean proportion of complications (**a**-**f**), 95% confidence interval and the relative weight of the individual studies. The diamond indicates the pooled estimate and its 95% confidence interval. The red bar indicates the 95% prediction interval. Prediction intervals illustrate which range of true effects expected to occur in similar studies in future settings

It was not possible to perform a meta-analysis for functional outcomes. A meta-analysis was performed for 6 different complications. Data from 10 studies were used to evaluate nonunion followed by data from 7 studies for infection. Seven studies reported hardware irritation, soft tissue problems [15, 17, 19–21, 23, 24] and hardware failure [15–17, 20, 22–24]. Four studies were included in a meta-analysis for persistent pain. (Fig. 1) The highest pooled incidences were found for complications hardware irritation (22, 95%CI 13–35 in 253 clavicles), soft tissue problems (9, 95%CI 6–13 in 207 clavicles) and infection (9, 95%CI 5–16 in 287 clavicles). A pooled incidence of unspecified persistent pain was reported in 6% (95%CI 2–20 in 172 clavicle) of cases. The pooled incidence of hardware failure and nonunion was 6% (95%CI 3–10 in 216 clavicles) and 3% (95%CI 1–8 in 337 clavicles) respectively.

The confidence in the estimates from the meta-analyses according to GRADE ranged between low and very low (Table 4 and Additional file 3).

**Table 4:** Summary of findings table including GRADE

Device	Outcome	No. of Studies	No. of Clavicles	Effect estimate (95%CI))	Quality of evidence (GRADE)
**Rockwood Pin & Hagie Pin**					
	Hardware Irritation	7	253	0.22 (0.13–0.35)	⨁⨁⨀⨀ LOW
	Infection	7	287	0.09 (0.05–0.16)	⨁⨁⨀⨀ LOW
	Soft Tissue Problems	7	207	0.09 (0.06–0.13)	⨁⨁⨀⨀ LOW
	Pain	4	172	0.06 (0.02–0.20)	⨁⨀⨀⨀ VERY LOW
	Hardware Failure	7	216	0.06 (0.03–0.10)	⨁⨁⨀⨀ LOW
	Nonunion	6	191	0.00 (0.00–0.04)	⨁⨁⨀⨀ LOW
	Scar Numbness	4	173	0.05 (0.02–0.09)	⨁⨀⨀⨀ VERY LOW
	Delayed Union	4	166	0.02 (0.01–0.06)	⨁⨀⨀⨀ VERY LOW
**TEN**					
	CMS	29	1270	94.40 (93.43–95.37)	⨁⨁⨁⨁ HIGH
	DASH	15	647	4.65 (2.61–6.68)	⨁⨁⨁⨁ HIGH
	Hardware Irritation	30	1273	0.20 (0.14–0.26)	⨁⨁⨁⨀ MODERATE
	Protrusion	25	1105	0.12 (0.08–0.18)	⨁⨁⨁⨀ MODERATE
	Malunion	3	193	0.07 (0.04–0.11)	⨁⨁⨀⨀ LOW
	Soft Tissue Problems	8	406	0.04 (0.03–0.08)	⨁⨀⨀⨀ VERY LOW
	Pain	3	136	0.04 (0.02–0.09)	⨁⨀⨀⨀ VERY LOW
	Nonunion	36	1436	0.03 (0.02–0.04)	⨁⨁⨁⨀ MODERATE
	Hardware Failure	19	800	0.03 (0.02–0.05)	⨁⨁⨀⨀ LOW
	Delayed Union	6	265	0.03 (0.02–0.06)	⨁⨀⨀⨀ VERY LOW
	Infection	29	1084	0.02 (0.01–0.03)	⨁⨁⨁⨀ MODERATE
**Sonoma CRx**					
	CMS	5	167	94.03 (92.31–95.76)	⨁⨁⨁⨀ MODERATE
	DASH	3	99	9.16 (3.94–14.37)	⨁⨁⨁⨀ MODERATE
	Cosmetic Dissatisfaction	3	92	0.06 (0.02–0.17)	⨁⨀⨀⨀ VERY LOW
	Hardware Failure	6	191	0.04 (0.02–0.08)	⨁⨁⨀⨀ LOW
	Infection	6	191	0.03 (0.01–0.07)	⨁⨁⨀⨀ LOW
	Nonunion	6	191	0.00 (0.00–0.04)	⨁⨁⨀⨀ LOW
**Threaded Pin**					
	Infection	3	106	0.01 (0.00–0.64)	⨁⨀⨀⨀ Very Low

**GRADE Working Group grades of evidence**

**High certainty:** We are very confident that the true effect lies close to that of the estimate of the effect

**Moderate certainty:** We are moderately confident in the effect estimate: The true effect is likely to be close to the estimate of the effect, but there is a possibility that it is substantially different

**Low certainty:** Our confidence in the effect estimate is limited: The true effect may be substantially different from the estimate of the effect

**Very low certainty:** We have very little confidence in the effect estimate: The true effect is likely to be substantially different from the estimate of effect

### Studies concerning the titanium elastic nail (TEN)

The first reports on using TEN in the treatment of DMCF dated from 2002 [35]. TENs with a diameter varying between 2 and 3.5 mm were used. Closed reduction rates were reported in 28 of 35 studies. The rates ranged from 15% [46] to 93% [27]. Most studies report a routine removal of the TEN in all cases mostly through a second surgical intervention but also removal under local anesthesia was described. The earliest routine nail removal was performed at 3 months [56] and the latest on average at 8.8 months [25].

#### Meta-analysis:

**Fig. 2:**
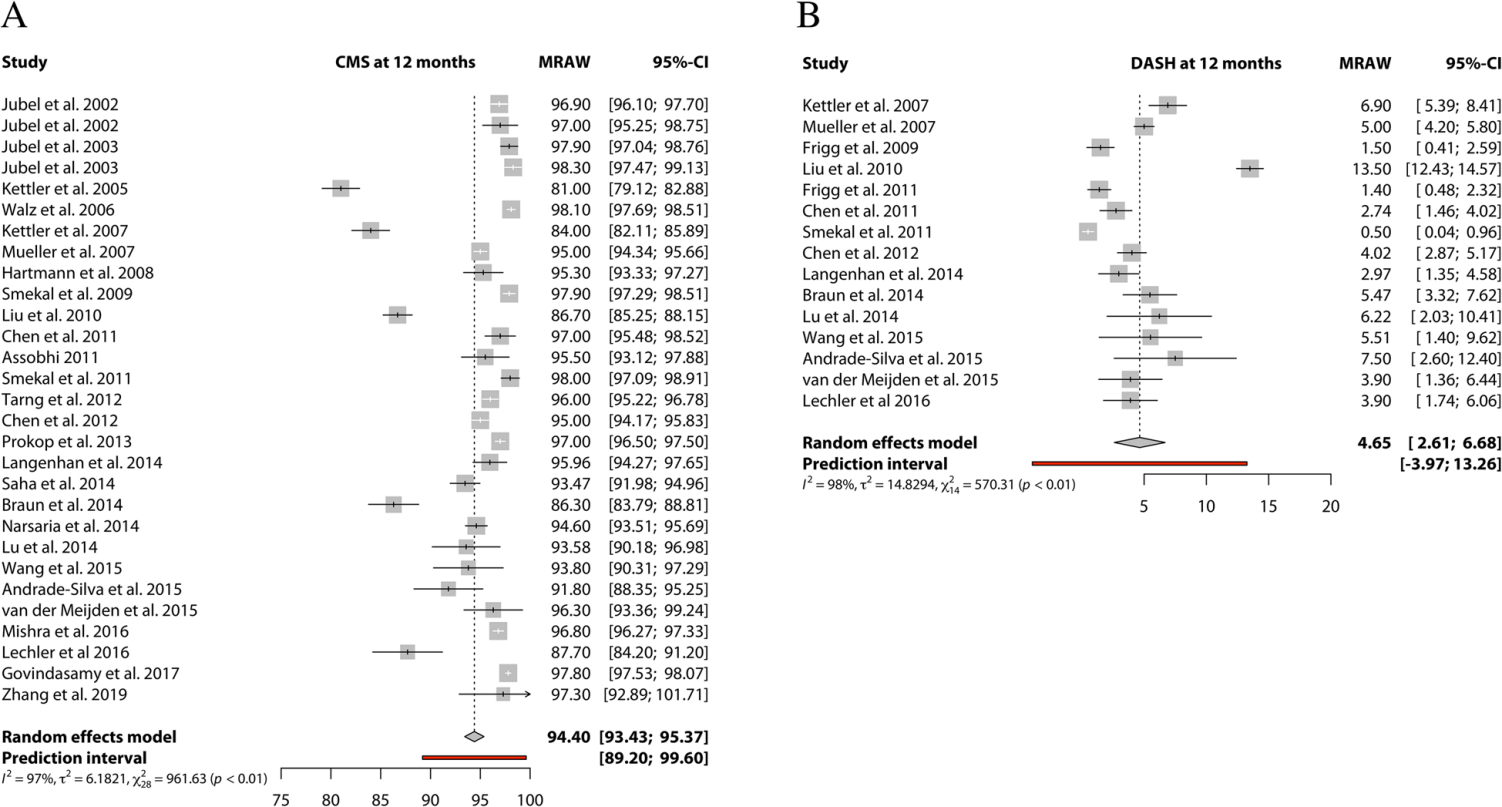
Forest plots of the included studies using the Titanium Elastic Nail reporting on (**a**) Constant-Murley score at 12 months, and (**b**) DASH score at 12 months. 95% confidence interval and the relative weight of the individual studies. The diamond indicates the pooled estimate and its 95% confidence interval. The red bar indicates the 95% prediction interval. Prediction intervals illustrate which range of true effects expected to occur in similar studies in future settings

A meta-analysis was performed for functional outcomes based on 30 studies reporting the Constant-Murley Score and 15 studies reporting a DASH score. (Fig. 2) The pooled data for the Constant-Murley score and DASH score at 12 months is 94.4 (95%CI 93.4–95.4 in 1290 clavicles) and 4.6 (95%CI 2.6–6.7 in 647 clavicles), respectively (Fig. 2). The confidence in the estimates from the meta-analyses according to GRADE concerning the functional outcomes were considered high due to the consistency and precision of the data in combination with the large number of clavicles involved (Table 4 and Additional file 3). The functional outcomes of two studies were not included in the meta-analysis [28, 31]. Fuglesang et al. [28] report the Constant-Murley and DASH scores of 60 TENs only by means of a line graph and van der Meijden et al. [31] report in-text Constant-Murley scores at 1 year follow up that differ from the line graph displayed. Visual evaluation of the line graphs however seems similar to the pooled incidences from the meta-analysis.

**Fig. 3:**
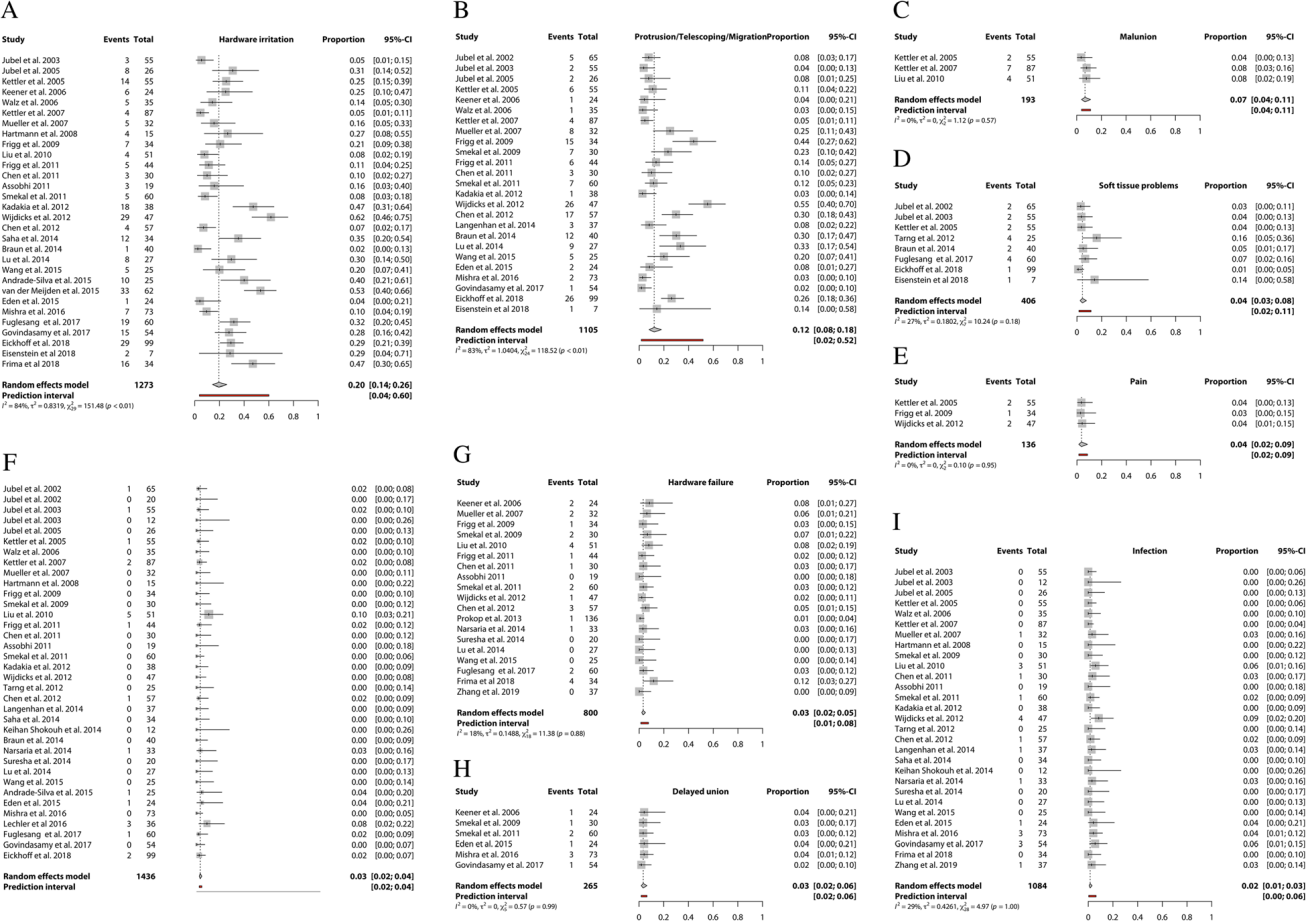
Forest plots of the included studies using the Titanium Elastic Nail reporting on (**a**) hardware irritation, (**b**) protrusion/telescoping/migration, (**c**) malunion, (**d**) soft tissue problems, (**e**) pain, (**f**) nonunion, (**g**) hardware failure, (**h**) delayed union, and (**i**) infection. Forest plots display the mean proportion of complications (A-H), 95% confidence interval and the relative weight of the individual studies. The diamond indicates the pooled estimate and its 95% confidence interval. The red bar indicates the 95% prediction interval. Prediction intervals illustrate which range of true effects expected to occur in similar studies in future settings

Data from 43 studies were pooled in the meta-analysis for evaluating complications rates using the TEN. Twenty-nine studies reported on infection, 29 studies on hardware irritation, 25 studies on protrusion/telescoping/migration, 19 on hardware failure, 12 on nonunion, 8 on soft tissue problems, 5 on malunion and 3 on pain. (Fig. 3) The two most common complications reported, protrusion/telescoping/migration and hardware irritation, are implant-related. The pooled incidence was 12% (95%CI 8–18 in 1105 clavicles) and 20% (95%CI 14–26 in 1273 clavicles), respectively.

Malunion after surgical management by means of a TEN was reported in 7% (95%CI 4–11 in 193 clavicles) and hardware failure was 3% (95%CI 2–5 in 800 clavicles). Pooled infection incidence was 2% (95%CI 0–3 in 1084 clavicles) and the pooled incidence of a nonunion using a TEN was 3% (95%CI 2–4 in 1436 clavicles). The confidence in the estimates from the meta-analyses according to GRADE concerning the functional outcomes ranged from moderate to very low (Table 4 and Additional file 3).

### Studies concerning the Sonoma CRx

#### Meta-analysis

**Fig. 4:**
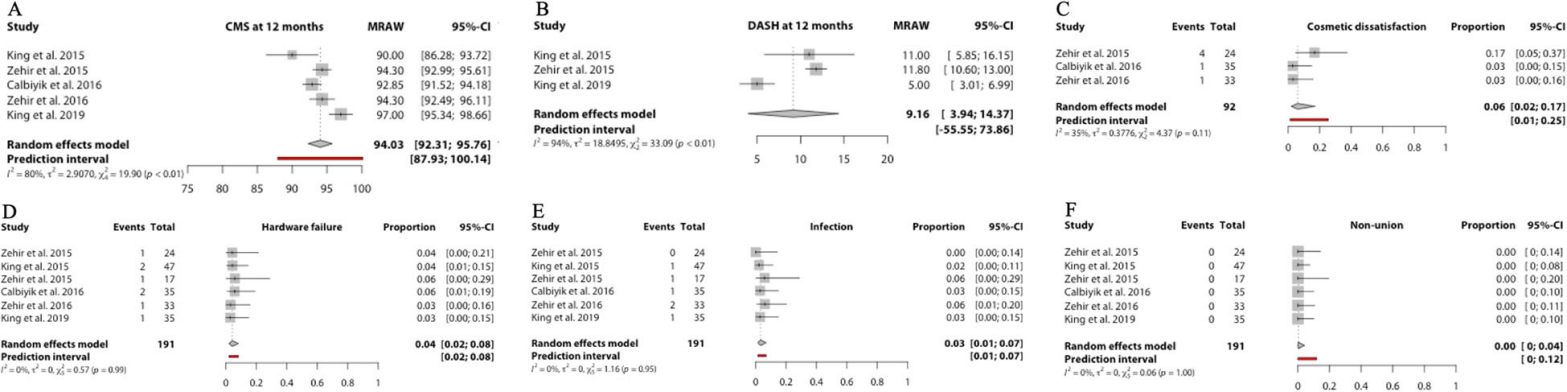
Forest plots of the included studies using the Sonoma CRx reporting on (**a**) Constant-Murley score at 12 months, (**b**) Disabilities of Arm, Shoulder and Hand Score at 12 months, (**c**) cosmetic dissatisfaction, (**d**) hardware failure, (**e**) infection, and (**f**) nonunion. Forest plots display the mean functional outcome (**a** and **b**) or proportion of complications (**c**-**f**), 95% confidence interval and the relative weight of the individual studies. The diamond indicates the pooled estimate and its 95% confidence interval. The red bar indicates the 95% prediction interval. Prediction intervals illustrate which range of true effects expected to occur in similar studies in future settings

Six studies were included in the meta-analysis. Data from 5 studies were pooled for functional outcomes using the Constant-Murley score. The pooled Constant-Murley score at 12 months was 94.0 (95%CI 92–96 in 167 clavicles). Six studies reported on nonunion, infection and hardware failure. Three studies reported cosmetic dissatisfaction. (Fig. 4) The pooled incidence for cosmetic dissatisfaction was highest at 6% (95%CI 2–17 in 92 clavicles), followed by of hardware failure (4%; 95%CI 2–8 in 191 clavicles) and infection (3%; 95%CI 1–7 in 191 clavicles). No reports of non-union using the Sonoma CRx were reported, the pooled incidence was 0% (95%CI 0–4 in 191 clavicles).

Two studies reported on persistent pain as a complication [68, 71] and 1 study mentions the occurrence of a delayed union [67].

The confidence in the estimates from the meta-analyses according to GRADE concerning the functional outcomes were considered moderate. Although the results were consistent, the data originate from very limited group of authors. The confidence in the other meta-analyses according to GRADE were low to very low (Table 4 and Additional file 3).

### Studies concerning a threaded elastic nail

Meta-analysis was only possible for infection [73–75] and the pooled incidence was 5% (95%CI 1–34 in 106 clavicles).

The confidence in the estimates from this meta-analysis according to GRADE was very low (Table 4 and Additional file 3: Additional file 3). Other complications described for this type of fixation were soft tissue problems, delayed union and malunion. (Table 2).

### Studies concerning the Knowles pin

One study reported 4 hardware irritations in 56 patients [77] and another study reported a nonunion rate of 5.6% [79]. No meta-analysis was possible for this device type.

### Study concerning a second generation TEN

One level IV study described the results of a second generation TEN in 36 patients [80]. It reported a Constant-Murley score of 93.4 (SD2.7) and 3 complications; 2 protrusions and 1 hardware irritation.

### Sensitivity analysis

The sensitivity analysis including only studies with a low risk of bias showed our results to be robust. The complete results of the sensitivity analysis can be found in Additional file 4.

### Publication bias

In those cases that publication bias could be assessed, its presence was unlikely based on the inspection of the funnel plots and evaluation of Egger’s or Peters’ tests. Only for the Constant Murley and DASH scores the tests for funnel plot asymmetry were significant, but publication bias seems unlikely here due to ceiling effects in both scores.

## Discussion

In this study the functional outcomes and complications after surgical treatment of DMCF with an intramedullary device were systematically reviewed. Good functional results and union rates irrespective of the type of device are found in the reviewed literature. However, there are clear device-related and device-specific complications for each. The pooled Constant-Murley scores of the TEN and Sonoma CRx were 94.4 (95%CI 93–95) and 94.0 (95%CI 92–96), respectively. Since the Constant-Murley score ranges from 0 to 100 points and higher scores are better, the pooled scores can be considered good. Though the minimally clinical important difference (MCID) for both the Constant-Murley score is unknown for midshaft clavicular fractures in particular it is described that the MCID in Constant Murley scores for shoulder pathology is 10.4 points [81]. Therefore, with an SD reported well within that range our conclusion seems valid as is the confidence in the estimate according to GRADE. The pooled DASH score for the TEN was 4.6 (95%CI 2.6–6.7). The functional outcomes for the Rockwood/Hagie pin could not be analyzed because all identified papers reported different functional outcome measures. This study supports the need for uniform reporting of functional outcomes and in the case of clavicle fracture treatment the Constant-Murley and the DASH are the ones most commonly used.

The most commonly reported complications after intramedullary fixation of DMCFs are implant-related and implant-specific complications. For the TEN, hardware irritation, protrusion, telescoping and migration, are major contributors to the total complication rate. The explanation for this finding may be that the TEN re-aligns but does not fixate in both fracture elements of the DMCF. These TEN-specific complications lead to infection, soft-tissue problems, pain, early re-interventions (removal or additional cutting of the nail) and loss of reduction with subsequent secondary shortening. When using the Rockwood/Hagie Pin, pooled incidence of hardware irritation was 22% (95%CI 13–35). This may be explained by the two bulky nuts at the posterolateral aspect of the clavicle where the pin is inserted and is has been reported to be an important disadvantage of the implant [15, 19, 22]. For the Sonoma CRx no reports on hardware irritation were found since this device has no extra-cortical prominences and is fully embedded in the clavicular cortex.

With regards to the TEN, there is a pooled malunion incidence of 7% (95%CI 4–11). Reports on persistent average shortening after union range between 3.5 and 6.3 mm [27, 37, 54]. Others report on shortening after union of more > 1 cm in 2.3–50% of cases [41, 57, 60]. Since shortening of the DMCF can lead to post-traumatic symptoms, altered scapular kinematics and the occurrence of gleno-humeral joint arthritis, shortening is an important issue to prevent and could be interpreted as a disadvantage of this intramedullary fixation device.

There are no studies specifically reporting on the presence or absence of post-operative shortening after fracture fixation with the Sonoma CRx. Concerning the Rockwood pin only Mudd et al. [21] reports a secondary shortening of 4-7 mm in 22% of patients which all occurred after early pin removal due to complications.

The pooled incidence for infection was 9% (95%CI 5–16) when using the Rockwood/Hagie pin, 3% (95%CI 1–7) when using the Sonoma CRx and 2% (95%CI 0–3) with use of the TEN. The two postero-lateral nuts that can cause wound-breakdown and subsequent infection may explain the high infection rate of the Rockwood/Hagie pin.

Hardware failure was 6% (95%CI 3–10) for the Rockwood/Hagie Pin compared to 3% (95%CI 2–5) for TEN and 4% (95%CI 2–8).

Meta-analysis shows nonunion incidences to be similar between the Rockwood/Hagie pin (3%;95%CI 1–8) and to 3% (95%CI 2–4) with the use of the TEN. The pooled incidence of nonunion for the Sonoma CRx was 0% (95%CI 0–4). Although no non-unions were reported in the Sonoma CRx group the confidence this outcome according to GRADE was low due to the limited number of clavicles included and the select group of authors introducing the risk of bias.

This systematic review furthermore identified the common denominator amongst many authors that routine removal of hardware is not considered a complication. However, a case could be made that every secondary intervention including hardware removal is an additional procedure which subjects the patient to associated morbidity and costs and therefore is not desirable.

As for all systematic reviews this study is limited by the quality of evidence available. In most meta-analyses of reported complications the evidence was graded as low to very low. Furthermore, only studies written in English, German or Dutch were included in this systematic review which could be a potential limitation of this study. Complications and early re-interventions are reported in some studies, [21, 33–35, 51, 54, 57] but underreporting is very likely to occur. Most studies do not clearly report causes for implant failure, measures taken with occurrence of infection or information concerning implant migration or secondary shortening. Only few specifically report on the presence or absence of certain relevant complications such as secondary shortening, neuropathy of the supraclavicular nerve, delayed union and persistent pain. This information could be interesting to fully report in future studies and is a limitation of this review. Another limitation is that not all functional outcomes and complications were reported in a similar manner leading to heterogeneity of the various studies. To account for the expected heterogeneity, a random effects model was used. In the case of functional outcome scores for TEN and Sonoma the confidence in the estimates was high and moderate, respectively. Lastly, the follow up differed between studies ranging from 3 months to 7 years. This may have resulted in differences in reporting of complications and functional outcomes. Although most complications would likely occur within the first 3 months this could lead to underreporting this could further negatively influence the confidence in the estimates reported.

In the last years multiple meta-analysis comparing the gold standard of plate fixation and intramedullary devices (irrespective of device or plate type) for the management of midshaft clavicle fractures have been published [82–89]. These studies report similar [82–84, 86–88] or superior [85, 89] functional outcomes and union rates in the intramedullary fixation group. Furthermore, most report a higher rate of complications (such as infection, refracture rate) and increased surgical time when using plate fixation, making an evaluation of the devices described in the present study even more relevant [82, 83, 86–89].

The results of this systematic review show there is still room for improvement in treating DMCF in an intramedullary fashion. For newer designs it may be interesting to take the implant-related and implant-specific complications described in this systematic review into account in order to optimize future treatment strategies.

## Conclusion

Although most studies were of low quality, in general, good functional results and union rates irrespective of the type of device are found in the reviewed literature. However, there are clear device-related and device-specific complications for each. The results of this systematic review and meta-analysis can help guide surgeons in choosing the appropriate operative strategy, implant and informing their patients.

## Supplementary information

**Additional file 1.** Search strategy.

**Additional file 2.** PRISMA Flow Diagram.

**Additional file 3.** GRADE Assessment.

**Additional file 4.** Sensitivity analysis Low Risk Studies using Random Effects Model.

## Data Availability

The detailed search strategy for this systematic review is available in Additional file 2. The review protocol adhered to by the authors is available via PROSPERO (CRD42018086518). The PRISMA flowchart is available in Additional file 1. Additional file 3 and Additional file 4 contain the GRADE assessment by domain and the sensitivity analysis.

## Abbreviations

ASES: American Shoulder Elbow Surgeons
CI: Confidence interval
DASH: Disabilities of arm shoulder hand
DMCF: Displaced mid-shaft clavicle fractures
FL: Florida
IN: Indiana
LoE: Level of evidence
MCID: Minimally clinical important difference
MI: Michigan
ORIF: Open reduction internal fixation
PRISMA: Preferred reporting items for systematic reviews and meta-analyses
TEN: Titanium elastic nail
USA: United States of America
