# Supplementary material for: Functional outcomes and complications of intramedullary fixation devices for Midshaft clavicle fractures: a systematic review and meta-analysis
Source: BMC Musculoskelet Disord. 2020 Jun 22;21:395. doi: 10.1186/s12891-020-03256-8 (PMC7310279; doi:10.1186/s12891-020-03256-8)
Supplement: Supplementary file 1 — Additional file 1. Search strategy. [file 12891_2020_3256_MOESM1_ESM.docx]

**Appendix 1. Search strategy**

Last search was performed on 31 January 2020

**PubMed**

(((((((clavic*) OR midclavic*) OR "Clavicle"[Mesh])) AND ((fracture) OR "Fractures, Bone"[Mesh])) AND ((intramedullary) OR "Fracture Fixation, Intramedullary"[Mesh])) AND (((((nail) OR pin) OR rod) OR screw) OR "Bone Nails"[Mesh])) AND (((((((((complication) OR ("Intraoperative Complications"[Mesh] OR "Postoperative Complications"[Mesh]))) OR ((union) OR (("Fractures, Malunited"[Mesh]) OR "Fractures, Ununited"[Mesh]))) OR ((survival) OR "Survival Rate"[Mesh])) OR ((failure) OR "Prosthesis Failure"[Mesh])) OR ((safety) OR "Safety"[Mesh])) OR ((function) OR "Recovery of Function"[Mesh])) OR ((outcome) OR ("Patient Outcome Assessment"[Mesh] OR "Outcome Assessment, Health Care"[Mesh])))

157

**ScienceDirect**

title-abs-key(clavicle OR clavicular OR midclavicle OR midclaviclular OR clavicula) AND (fracture) AND intramedullary AND ((pin or rod) OR (nail or screw)) OR survival OR safety OR outcome OR function OR performance OR union)

160

**Embase**

((((((clavic*) OR midclavic*)) AND fracture*) AND intramedullary) AND ((((nail) OR pin) OR rod OR screw))) AND ((((((((complication) OR union) OR survival) OR performance) OR failure) OR safety) OR function) OR outcome

159

**Cochrane Library**

(((((((clavic*) OR midclavic*) OR "Clavicle"[Mesh])) AND ((fracture) OR "Fractures, Bone"[Mesh])) AND ((intramedullary) OR "Fracture Fixation, Intramedullary"[Mesh])) AND (((((nail) OR pin) OR rod) OR screw) OR "Bone Nails"[Mesh])) AND (((((((((complication) OR ("Intraoperative Complications"[Mesh] OR "Postoperative Complications"[Mesh]))) OR ((union) OR (("Fractures, Malunited"[Mesh]) OR "Fractures, Ununited"[Mesh]))) OR ((survival) OR "Survival Rate"[Mesh])) OR ((failure) OR "Prosthesis Failure"[Mesh])) OR ((safety) OR "Safety"[Mesh])) OR ((function) OR "Recovery of Function"[Mesh])) OR ((outcome) OR ("Patient Outcome Assessment"[Mesh] OR "Outcome Assessment, Health Care"[Mesh])))

46

**Clinical trial registries**

ClinicalTrials.gov, controlled-trials.com (ISRCTN), Australian New Zealand Clinical Trials Registry (ANZCTR), Chinese Clinical Trial Registry (CCTR), EU Clinical Trials Register (EU-CTR), and The Netherlands National Trial Register (NTR) were searched using the following keywords:

Clavicle

8
