# Supplementary material for: Functional outcomes and complications of intramedullary fixation devices for Midshaft clavicle fractures: a systematic review and meta-analysis
Source: BMC Musculoskelet Disord. 2020 Jun 22;21:395. doi: 10.1186/s12891-020-03256-8 (PMC7310279; doi:10.1186/s12891-020-03256-8)
Supplement: Supplementary file 2 — Additional file 2. PRISMA Flow Diagram. [file 12891_2020_3256_MOESM2_ESM.doc]

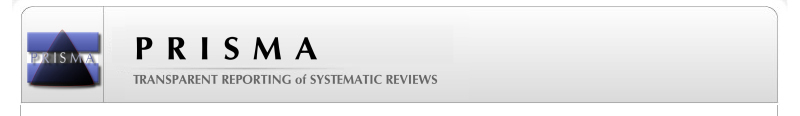
**PRISMA Flow Diagram**

**Screening**

**Included**

**Eligibility**

**Identification**

Records identified through database searching
(n = 530)

Additional records identified through other sources
(n = 0 )

Records after duplicates removed
(n = 368)

Records screened
(n = 368)

Records excluded
(n = 293)

Full-text articles assessed for eligibility
(n = 75)

Studies included in qualitative analysis
(n = 67)

Studies included in quantitative synthesis (meta-analysis)
(n = 62)

Full-text articles excluded, with reasons
(n = 8)

3 Duplicate Data Sets^1-3^

1 Interim Data Set^4^

1 No distinction within plate and IM group^5^

3 Unable to obtain full-text^6-8^
