# Supplementary material for: Functional outcomes and complications of intramedullary fixation devices for Midshaft clavicle fractures: a systematic review and meta-analysis
Source: BMC Musculoskelet Disord. 2020 Jun 22;21:395. doi: 10.1186/s12891-020-03256-8 (PMC7310279; doi:10.1186/s12891-020-03256-8)
Supplement: Supplementary file 3 — Additional file 3. GRADE Assessment. [file 12891_2020_3256_MOESM3_ESM.docx]

|  |  |  |  |  | **GRADE Domains** | | | | | | | |  |
| --- | --- | --- | --- | --- | --- | --- | --- | --- | --- | --- | --- | --- | --- |
| **Device** | **Outcome** | **No. of Studies** | **No. of Clavicles** | **Effect estimate (95%CI))** | **Risk of Bias** | **Inconsistency** | **Imprecision** | **Indirectness** | **Publication Bias** | **Large Magnitude of Effect** | **Dose Repsonse Gradient** | **Residual Confounding** | **Quality of evidence (GRADE)** |
|  |  |  |  |  |  |  |  |  |  |  |  |  |  |
| **Rockwood Pin & Hagie Pin** |  |  |  |  |  |  |  |  |  |  |  |  |  |
|  | Hardware Irritation | 7 | 253 | 0.22 (0.13 – 0.35) | x | x | x | NA | o | o | o | o | ⨁⨁⨀⨀ LOW |
|  | Infection | 7 | 287 | 0.09 (0.05 – 0.16) | x | x | x | NA | o | o | o | o | ⨁⨁⨀⨀ LOW |
|  | Soft Tissue Problems | 7 | 207 | 0.09 (0.06 – 0.13) | x | x | x | NA | o | o | o | o | ⨁⨁⨀⨀ LOW |
|  | Pain | 4 | 172 | 0.06 (0.02 – 0.20) | x | x | x | NA | o | o | o | o | ⨁⨀⨀⨀ VERY LOW |
|  | Hardware Failure | 7 | 216 | 0.06 (0.03 – 0.10) | x | x | x | NA | o | o | o | o | ⨁⨁⨀⨀ LOW |
|  | Nonunion | 6 | 191 | 0.00 (0.00 – 0.04) | x | x | o | NA | o | o | o | o | ⨁⨁⨀⨀ LOW |
|  | Scar Numbness | 4 | 173 | 0.05 (0.02 – 0.09) | x | x | x | NA | o | o | o | o | ⨁⨀⨀⨀ VERY LOW |
|  | Delayed Union | 4 | 166 | 0.02 (0.01 – 0.06) | x | x | x | NA | o | o | o | o | ⨁⨀⨀⨀ VERY LOW |
|  |  |  |  |  |  |  |  |  |  |  |  |  |  |
| **TEN** |  |  |  |  |  |  |  |  |  |  |  |  |  |
|  | CMS | 29 | 1270 | 94.40 (93.43 – 95.37) | x | o | o | NA | o | x | o | o | ⨁⨁⨁⨁ HIGH |
|  | DASH | 15 | 647 | 4.65 (2.61 – 6.68) | x | o | o | NA | o | x | o | o | ⨁⨁⨁⨁ HIGH |
|  | Hardware Irritation | 30 | 1273 | 0.20 (0.14 – 0.26) | x | o | x | NA | o | x | o | o | ⨁⨁⨁⨀ MODERATE |
|  | Protrusion | 25 | 1105 | 0.12 (0.08 – 0.18) | x | o | x | NA | o | x | o | o | ⨁⨁⨁⨀ MODERATE |
|  | Malunion | 3 | 193 | 0.07 (0.04 – 0.11) | x | x | x | NA | o | o | o | o | ⨁⨁⨀⨀ LOW |
|  | Soft Tissue Problems | 8 | 406 | 0.04 (0.03 – 0.08) | x | x | x | NA | o | o | o | o | ⨁⨀⨀⨀ VERY LOW |
|  | Pain | 3 | 136 | 0.04 (0.02 – 0.09) | x | x | x | NA | o | o | o | o | ⨁⨀⨀⨀ VERY LOW |
|  | Nonunion | 36 | 1436 | 0.03 (0.02 – 0.04) | x | o | x | NA | o | x | o | o | ⨁⨁⨁⨀ MODERATE |
|  | Hardware Failure | 19 | 800 | 0.03 (0.02 – 0.05) | x | x | x | NA | o | o | o | o | ⨁⨁⨀⨀ LOW |
|  | Delayed Union | 6 | 265 | 0.03 (0.02 – 0.06) | x | o | x | NA | o | o | o | o | ⨁⨀⨀⨀ VERY LOW |
|  | Infection | 29 | 1084 | 0.02 (0.01 – 0.03) | x | o | x | NA | o | x | o | o | ⨁⨁⨁⨀ MODERATE |
|  |  |  |  |  |  |  |  |  |  |  |  |  |  |
| **Sonoma CRx** |  |  |  |  |  |  |  |  |  |  |  |  |  |
|  | CMS | 5 | 167 | 94.03 (92.31 – 95.76) | o | x | o | NA | x | o | o | o | ⨁⨁⨁⨀ MODERATE |
|  | DASH | 3 | 99 | 9.16 (3.94 – 14.37) | o | x | o | NA | x | o | o | o | ⨁⨁⨁⨀ MODERATE |
|  | Cosmetic Dissatisfaction | 3 | 92 | 0.06 (0.02 – 0.17) | x | x | x | NA | x | o | o | o | ⨁⨀⨀⨀ VERY LOW |
|  | Hardware Failure | 6 | 191 | 0.04 (0.02 – 0.08) | o | o | x | NA | x | o | o | o | ⨁⨁⨀⨀ LOW |
|  | Infection | 6 | 191 | 0.03 (0.01 – 0.07) | x | o | x | NA | x | o | o | o | ⨁⨁⨀⨀ LOW |
|  | Nonunion | 6 | 191 | 0.00 (0.00 – 0.04) | x | x | o | NA | x | o | o | o | ⨁⨁⨀⨀ LOW |
|  |  |  |  |  |  |  |  |  |  |  |  |  |  |
| **Threaded Pin** |  |  |  |  |  |  |  |  |  |  |  |  |  |
|  | Infection | 3 | 106 | 0.01 (0.00 – 0.64) | x | x | x | NA | o | o | o | o | ⨁⨀⨀⨀ Very Low |
|  |  |  |  |  |  |  |  |  |  |  |  |  |  |

**x = Present, o = Not present, NA = Not Applicable**

**GRADE Working Group grades of evidence**

**High certainty:** We are very confident that the true effect lies close to that of the estimate of the effect

**Moderate certainty:** We are moderately confident in the effect estimate: The true effect is likely to be close to the estimate of the effect, but there is a possibility that it is substantially different

**Low certainty:** Our confidence in the effect estimate is limited: The true effect may be substantially different from the estimate of the effect

**Very low certainty:** We have very little confidence in the effect estimate: The true effect is likely to be substantially different from the estimate of effect
