# Supplementary material for: Functional outcomes and complications of intramedullary fixation devices for Midshaft clavicle fractures: a systematic review and meta-analysis
Source: BMC Musculoskelet Disord. 2020 Jun 22;21:395. doi: 10.1186/s12891-020-03256-8 (PMC7310279; doi:10.1186/s12891-020-03256-8)
Supplement: Supplementary file 4 — Additional file 4. Sensitivity analysis Low Risk Studies using Random Effects Model. [file 12891_2020_3256_MOESM4_ESM.docx]

**Supplement A. Sensitivity analysis Low Risk Studies using Random Effects Model.**

There were no low risk studies available for sensitivity analysis of: malunion TEN, pain TEN, delayed union TEN, infection Rockwood pin and hardware irritation Rockwood pin.

Only 1 low risk study was available for evaluating scar numbness Rockwood pin.

Similar pooled outcomes were calculated for all other meta-analysis.

| **Device type** | **Complication** | **Number of low risk studies** | **Pooled incidence** |
| --- | --- | --- | --- |
| **Sonoma CRx** |  |  |  |
|  | Cosmetic dissatisfaction | 2 | 8% (95%CI 2 - 24) |
|  | Hardware failure | 3 | 4% (95%CI 2 - 11) |
|  | Infection | 3 | 2% (95%CI 1 - 8) |
|  | Nonunion | 3 | 0%(95%CI 0-11) |
|  |  |  |  |
| **Rockwood/Hagie Pin** | |  |  |
|  | Non-union | 2 | 2% (95%CI 0 - 14) |
|  | Hardware failure | 2 | 4% (95%CI 1 – 16) |
|  |  |  |  |
| **TEN** |  |  |  |
|  | Hardware irritation | 12 | 23% (95%CI 15 – 33) |
|  | Protrusion/Telescoping/Migration | 9 | 10% (95%CI 5 – 17) |
|  | Soft tissue problems | 3 | 3% (95%CI 1 – 7) |
|  | Hardware failure | 8 | 5% (95%CI 3 – 8) |
|  | Infection | 10 | 3% (95%CI 1 – 5) |
|  | Non-union | 13 | 2% (95%CI 1 – 3) |
|  | Delayed union | 4 | 3% (95%CI 2 – 7) |
|  |  |  |  |
|  |  |  |  |
| **Device type** | **Functional Outcome Score** | **Number of low risk studies** | **Pooled incidence** |
| **Sonoma CRx** |  |  |  |
|  | CMS | 2 | 94.9 (95%CI 91 - 99) |
|  |  |  |  |
| **TEN** |  |  |  |
|  | CMS | 12 | 96.7 (95%CI 96 - 97) |
|  | DASH | 5 | 3.6 (95%CI 1 - 6) |
